# Interactions Between the Transcription Factor BOL/DRNL/ESR2 and the Jasmonate Pathway

**DOI:** 10.3390/plants14121757

**Published:** 2025-06-08

**Authors:** Beatriz E. Ruiz-Cortés, Yolanda Durán-Medina, C. Cecilia Ramos-Tamayo, Herenia Guerrero-Largo, Ma. Isabel Cristina Elizarraraz-Anaya, Omar Fabián Hernández-Zepeda, Enrique Ramírez-Chávez, Michiel Lammers, Ruud A. de Maagd, Jorge Molina-Torres, Stefan de Folter, Nayelli Marsch-Martínez

**Affiliations:** 1 Irapuato Unit, Biotechnology and Biochemistry Department, Cinvestav, Irapuato C.P. 36824, Guanajuato, Mexico; beatriz.ruiz@cinvestav.mx (B.E.R.-C.);; 2 Advanced Genomics Unit, Cinvestav, Irapuato C.P. 36824, Guanajuato, Mexico; 3 Genómica Alimentaria, Universidad de la Ciénega del Estado de Michoacán de Ocampo, Sahuayo C.P. 59103, Michoacán, Mexico; 4 Wageningen University & Research, P.O. Box 16, 6700 AA Wageningen, The Netherlands

**Keywords:** jasmonate, transcription factor, bolita, BOL, enhancer of shoot regeneration 2, ESR2, dornroschen-like, DRNL, Arabidopsis (*Arabidopsis thaliana*), development, stamen, callus formation

## Abstract

BOL/DRNL/ESR2, an AP2/ERF transcription factor, regulates early organ development in Arabidopsis (*Arabidopsis thaliana*). Its loss of function causes flower organ defects, while its overexpression induces green callus formation in roots without the addition of hormones. Jasmonates, plant hormones known as major players in stress responses, also regulate some aspects of organ development (e.g., stamen development and plant and organ growth). Here, we studied the interaction between BOL and the JA pathway. We found that exogenous application of methyl jasmonate (MeJA) partially rescued the stamen phenotypes in *bol-cr* mutants, linking BOL and JA-mediated stamen development. Moreover, MeJA treatments in wild-type plants partially mimicked some *bol-D* mutant phenotypes like reduced rosette and root size, while JA inhibition restored wild-type leaf curvature, suggesting an alteration in JA homeostasis in the gain-of-function mutant. *BOL* overexpression caused increased JA levels, whereas *bol* loss-of-function plants had reduced levels. Furthermore, inducible *BOL* activity led to downregulation of a JA-responsive marker. Finally, JA biosynthesis inhibition affected BOL-induced root callus formation and led to an expansion of the *BOL* expression domain in roots. Our findings indicate that BOL modulates parts of the JA pathway and that feedback from the JA pathway appears to affect expression of the transcription factor.

## 1. Introduction

In plants, the regulation of developmental processes involves transcriptional regulators and plant hormones, among other things [1]. One such transcriptional regulator is *BOLITA/DÖRNROSCHEN-LIKE/ENHANCER OF SHOOT REGENERATION 2* (*BOL/DRNL/ESR2*), a gene that codes for a 306-amino-acid protein that belongs to the AP2/ERF family of transcription factors [2]. It is expressed at the earliest stages of the developing aerial organs, and at later stages, *BOL* expression is still detectable in developing petals, stamens, and lateral regions of the developing gynoecium [2,3,4,5]. The overexpression of *BOL* in Arabidopsis (*Arabidopsis thaliana*) causes severe shoot and root growth inhibition. Moreover, it induces the formation of green calli in roots, which can form a new plant when they are separated from this organ. In tobacco, *BOL* overexpression causes the formation of ectopic floral organs, stunting, and curvature in the leaves [2]. Moreover, BOL/ESR2 enhances shoot regeneration [4]. The loss of *BOL/ESR2/DRNL* function has been characterized in the *drnl-2* mutant [5], which presents cotyledon fusions [6] and defects in the development of all floral organs, although the defects are more severe in the gynoecium and stamens [3,5,7]. Gynoecia in *drnl-2* plants are slightly longer than those in wild-type plants and frequently present asymmetric valves, or lack one valve [3,7]. The number and size of stamens of the *drnl-2* mutant are reduced compared to wild-type plants, and some of them are converted into filamentous structures [5]. While in Arabidopsis, the loss of *BOL/DRNL* function causes a partially penetrant phenotype of fused cotyledons [6], in tomato, the loss of function of the only ortholog of *BOL*, called *LEAFLESS* (*LFS*), produces pin-like shoots with active shoot apical meristems that are unable to form cotyledons or leaves. *LFS* expression was found in leaf primordia and axillary buds, consistent with its ortholog in Arabidopsis [8].

As a transcription factor, BOL/DRNL/ESR2 is expected to regulate the expression of multiple target genes associated with diverse processes. It has been reported to regulate *CUP-SHAPED COTYLEDON 1* (*CUC1*), a gene involved in meristem specification [4]. This was found through a microarray expression analysis using an *ESR2/BOL* inducible line, *35S::ESR2-ER*. The authors showed that the phenotypes of *ESR2/BOL*-induced expression decreased in the absence of *CUC1* [4]. Moreover, BOL/DRNL regulates family members of the transcriptional regulators *SHORT INTERNODES/STYLISH* (*SHI/STY*), which activate auxin biosynthesis [9]. Furthermore, BOL and its homolog DÖRNROSCHEN (DRN) control axillary meristem initiation through their interaction with the transcription factor REVOLUTA (REV), directly regulating the transcription of the *SHOOT MERISTEMLESS* (*STM*) gene [9]. Both microarray analyses [4] and RNA-seq studies [10] have identified genes such as *ARABIDOPSIS HISTIDINE PHOSPHOTRANSFER PROTEIN 6* (*AHP6)* as candidate targets acting downstream of DRNL/BOL. Recently, this transcription factor was found to interact with the Auxin Response Factor MONOPTEROS (MP) and to directly activate *AHP6* and *CYTOKININ OXIDASE 6* (*CKX6*) [11]. Moreover, BOL also activates *ISOPENTENYLTRANSFERASE 5* (*IPT5*) [12]. Other high-throughput analyses have found more candidate targets [10] in the context of founder cell expression [13]. As more candidate target genes are found, it seems that BOL/DRNL/ESR2 may act as a general coordinator of several processes.

Differential gene expression analyses between a BOL gain-of-function mutant and wild-type plants showed that many different processes were altered in the mutant, including hormonal pathways, and one of these was the jasmonate pathway [2,14]. This opened the question about a possible relation between the transcription factor and this hormonal pathway. Jasmonic acid (JA) and its derivatives, usually known as jasmonates (JAs), are lipid-derived signaling molecules synthesized from α-linolenic acid via the lipoxygenase pathway [15,16,17,18,19,20,21,22,23,24]. Upon stress or developmental signals, JAs accumulate and are perceived by the CORONATINE INSENSITIVE1 (COI1) receptor, triggering degradation of *JASMONATE-ZIM DOMAIN* (JAZ) repressor proteins, which release transcription factors like MYC2. These transcription factors activate jasmonate-responsive genes. Among them, MYC2 activates *JAZ* genes, causing a negative feedback loop in the JA signaling cascade [16,21,25,26,27,28].

Jasmonates have been extensively studied as signaling molecules that participate in the plant response to biotic and abiotic stresses, but they also play an important role as regulators of diverse development processes [28]. One of the functions attributed to jasmonates is as promoters of reproductive organ development. Mutants in JA biosynthesis and perception genes, such as *aos*, *opr3*, or *coi1*, exhibit male infertility. Their stamens show delayed anther dehiscence, their filaments do not elongate enough to reach the level of the stigma, and pollen viability is severely reduced [24,28,29]. Jasmonates have also been found to be regulators of plant growth. Moreover, increased levels of JA in the plant reduced its growth by 50% due to an inhibition of mitosis that caused a decrease in cell number [30]. Furthermore, the exogenous application of methyl jasmonate (MeJA) reduced the growth of the main root by 50% [31] due to the inhibition of the cell cycle [32].

Recently, it has been found that JAs play a significant role in promoting root regeneration. Treating plants with MeJA promoted division in normally inactive quiescent center cells and sped up the replacement of columella stem cells when they were damaged [32], while treatment with a JA receptor inhibitor greatly reduced root formation [33]. Similarly, JAs act as signals for root regeneration from leaf explants [34].

Interestingly, in *in vitro* plant cultures, JAs have been found to both inhibit but also to promote organogenesis and organ growth, depending on their concentration, tissue, species, and combination with other plant growth regulators [35].

Given the enrichment in JA genes among the differentially expressed genes in the *bol-D* activation tagging mutant and the similarities of some of the reported effects of JA and BOL phenotypes, we sought to explore the relationship between this transcription factor and the jasmonate pathway in the context of plant development.

## 2. Results

### 2.1. Gene-Edited bol-cr Mutant Alleles Show Stamen, Gynoecium, and Cotyledon Defects

**Figure 1:**
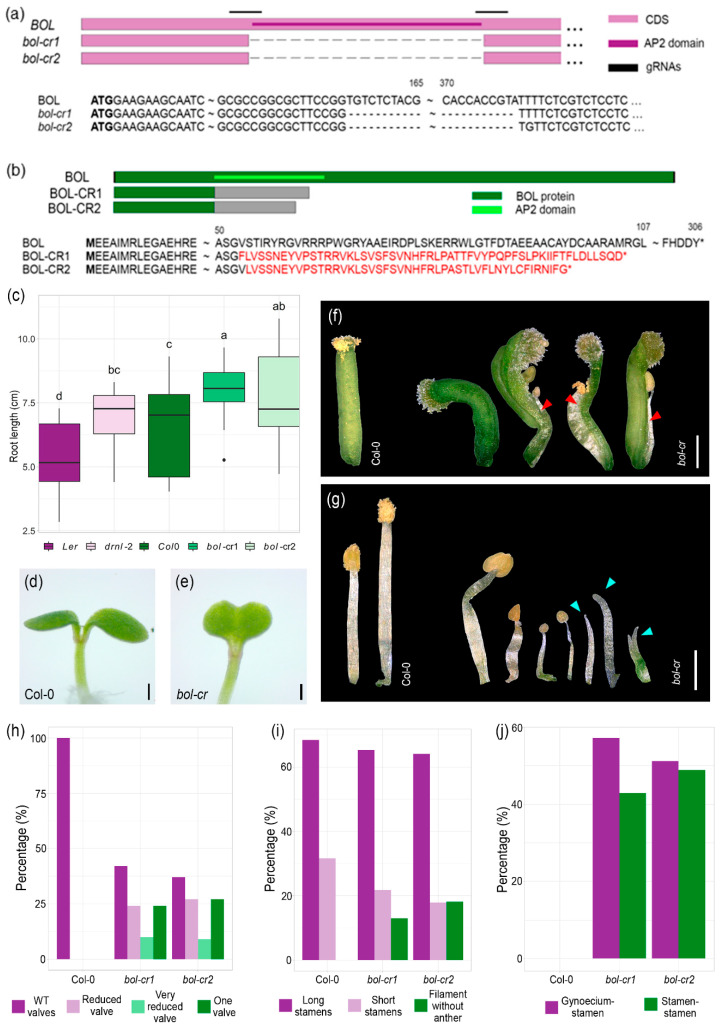
Genomic and protein structure of mutations generated by genomic editing and edited mutant phenotypes. (**a**,**b**) Genomic (**a**) and protein structure (**b**) of the CRISPR-Cas9-generated *BOL* mutations. Letters in red represent amino acids that are different from the original BOL protein. Asterisks represent stop codons. The dark green region represents the BOL protein, the light green region the AP2 domain within the protein, and the gray sections represent a region that, if translated, would produce a sequence different from the original BOL sequence. (**c**) Comparison of root length between 14-day-old wild-type and edited bol mutant (*bol-cr*) plants. ANOVA *p*-value = 3.58 × 10^−6^. Letters represent different statistical groups (Least Significant Difference, LSD test). (**d**) Representative 2-day-old Col-0 seedling phenotype. (**e**) Representative 2-day-old *bol-cr* mutant seedling phenotype showing fusion between cotyledons. (**f**) Representative gynoecium phenotypes of Col-0 wild-type and *bol-cr* mutants. The images show phenotypes observed in both *bol-cr* mutant lines. Red arrows indicate fusions between stamen and gynoecium. (**g**) Representative stamen phenotypes of Col-0 wild-type and *bol-cr* mutants. The images show phenotypes observed in both mutant lines. Blue arrows indicate filaments (stamens without anthers). (**h**) Frequencies of different gynoecium phenotypes of Col-0 and *bol-cr* mutants. (**i**) Frequencies of stamen phenotypes of Col-0 and *bol-cr* mutants. (**j**) Frequencies of type of fusion in flower organs of Col-0 and *bol-cr* mutants. Scale bars = 200 µm (**d**,**e**); 500 µm (**f**,**g**).

The *BOL* loss-of-function *drnl-2* mutant allele [5] was generated in the L*er* ecotype background and contains a missense mutation (A93V) in the AP2 domain. We sought to generate a new *BOL* mutant allele in the Col-0 ecotype to facilitate analyses of crosses to marker and mutant lines, many of which have been obtained in this ecotype. We also aimed to generate a new allele in which the whole AP2 domain was removed, to facilitate genotyping. For this, we used the CRISPR-Cas9 system to create a deletion in the AP2 domain of *BOL* in Col-0 plants. We designed two guide RNAs (gRNAs) that targeted the borders of the AP2 DNA binding domain in *BOL* (Figure 1a). We obtained 50 transformants in the T0 generation. T1 and T2 progeny plants were genotyped by PCR, and we found two independently edited homozygous mutant plants, named *bol-cr1* and *bol-cr2*. Sequencing showed that *bol-cr1* and *bol-cr2* contain a deletion of 224 bp between the two gRNAs, resulting in the complete removal of the AP2 domain and a shift in the reading frame in the sequence remaining after the deletion. If translated, these transcripts would result in chimeric, shorter proteins (107 amino acids for *bol-cr1* and 99 amino acids for *bol-cr2*), where the first 52 (*bol-cr1*) and 53 (*bol-cr2*) amino acids would be preserved, but the following amino acids would be different from the original BOL sequence (Figure 1a,b and Figure S1). We then allowed the mutations to segregate from the original T-DNA and confirmed, by PCR, that both mutant lines did not contain the transgene with the CRISPR-Cas9 machinery anymore.

*bol-cr1* and *bol-cr2* plants exhibited the same visible phenotype as the *drnl-2* mutant phenotype. All the mutants (*drnl-2*, *bol-cr1*, and *bol-cr2*) presented longer roots than their wild-type backgrounds (L*er* and Col-0) at 14 days after germination (Figure 1c). Both CRISPR-Cas9 mutants presented cotyledon fusion in about 4% of the plants (Figure 1d,e; *n* = 100), which is comparable to the *drnl-2* mutant [6]. They also exhibited the range of altered gynoecia phenotypes previously reported for the *drnl-2* mutant, including wild-type-like gynoecia, gynoecia with reduced valve sizes, and gynoecia with a single valve, as described in [3] and not observed in wild-type plants (Figure 1f,h). The stamens in the flowers of both BOL CRISPR-Cas9 mutants also showed altered phenotypes, similar to those reported for the *drnl-2* mutant [5]. Stamen phenotypes ranged from long stamens that were the same or almost the same size and shape as the wild type to shorter stamens with smaller anthers than the wild type and stamens that did not develop anthers and only consisted of a filament (Figure 1g,i). Moreover, *bol-cr* mutants presented the characteristic phenotype of fusion between organs, especially between the gynoecium and anthers, in approximately 35% of the flowers (Figure 1j, arrows in Figure 1f). Since the phenotypes were comparable to the *drnl-2* mutant phenotype, we concluded that the *bol-cr* mutants were suitable to study the loss of *BOL* function. As both *bol-cr* mutants presented the same phenotype, we used the *bol-cr2* allele for further experiments.

### 2.2. Exogenous Application of MeJA to Bol Loss-of-Function Mutants Partially Recovers the Wild-Type Stamen Phenotype

To evaluate whether the anther phenotypes of *bol* loss-of-function mutant flowers could be due, at least partially, to a deficiency of jasmonates, we analyzed the effect of MeJA applications on *bol-cr2* mutant flowers.

As the mutant presented variable stamen phenotypes, we counted the three different types of previously observed staminoid structures in control flowers and flowers that were treated with 50 and 100 μM MeJA: long stamens, short stamens, and filaments.

**Figure 2:**
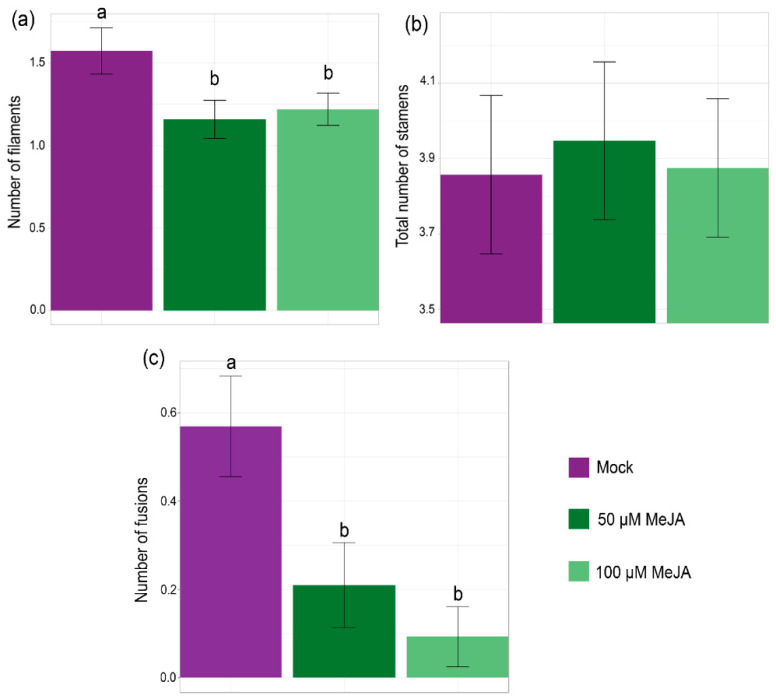
MeJA exogenous application to *bol* loss-of-function mutant flowers. (**a**) Number of filaments (stamens without anthers) per *bol-cr2* flower, mock or treated with 50 and 100 µM MeJA. *n* = 20–25 flowers, Kruskal–Wallis *p*-value = 0.042. Letters represent different statistical groups (Dunn’s test). (**b**) Total number of stamens (long, short, and filaments) per *bol-cr2* flower, mock or treated with 50 and 100 µM MeJA. *n* = 20–25 flowers, Kruskal–Wallis *p*-value = 0.965. (**c**) Number of floral organ fusions per *bol-cr2* flower mock or treated with 50 and 100 µM MeJA. *n* = 20–25 flowers, Kruskal–Wallis *p*-value = 0.0003. Letters represent different statistical groups (Dunn’s test).

We observed a reduction in the number of filaments in the flowers treated with MeJA (Figure 2a) while the total number of all staminoid structures remained unchanged (Figure 2b). Surprisingly, we also observed fewer fusions between organs in the flowers of the plants that were treated with MeJA in comparison to control flowers (Figure 2c). Therefore, the exogenous MeJA application could partially recover the wild-type stamen phenotype in the *bol-cr2* mutant. This suggested that the floral organ defect phenotype could be, at least in part, due to an alteration in the JA pathway.

### 2.3. MeJA Application or Inhibition Mimic or Partially Restore Bol-D Phenotypes

The activation-tagged gain-of-function mutant *bol-D* exhibits both reduced root growth and plant size [2]. Since JA applications also cause root growth inhibition [31,36] and rosette size reduction [30,32], we compared wild-type plants (Wassilewskija, Ws, the background ecotype of the *bol-D* mutant) treated with MeJA to *bol-D* plants.

**Figure 3:**
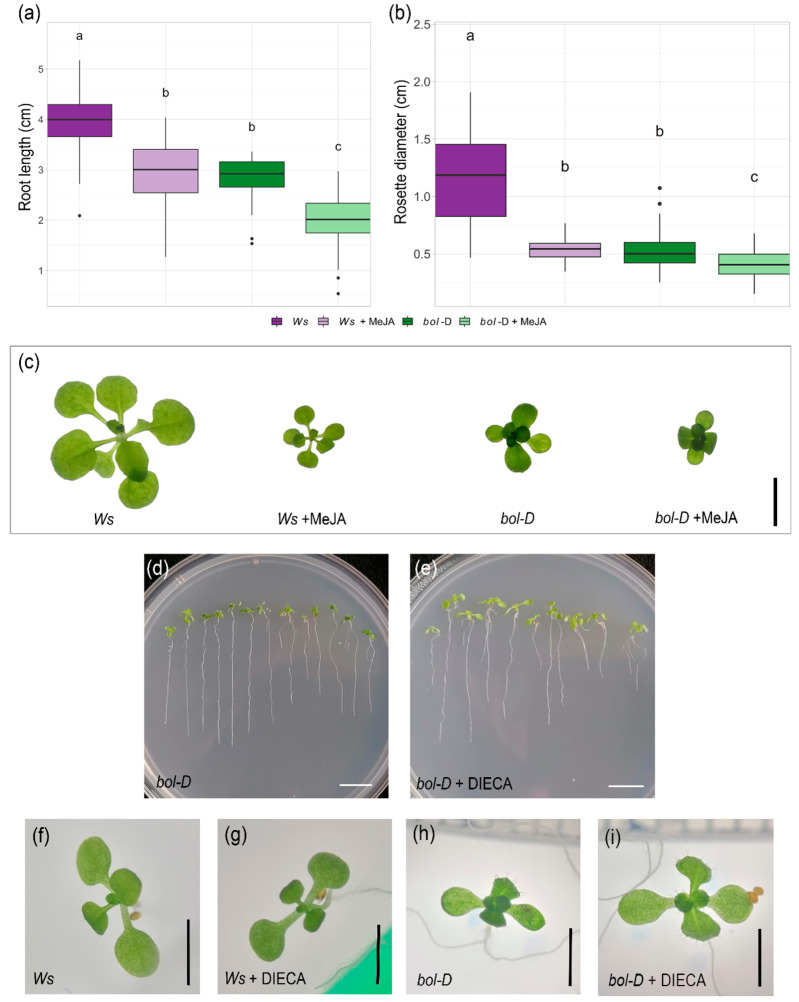
MeJA application or inhibition in wild-type and *bol-D* plants. (**a**) Root length of 9-day-old *bol-D* and Ws plants treated with 50 µM MeJA. (**b**) Rosette diameter of 9-day-old *bol-D* and Ws plants treated with 50 µM MeJA. (**c**) Rosette phenotype of 9-day-old *bol-D* and Ws plants treated with 50 µM MeJA. (**d**) 6-day-old *bol-D* plants. (**e**) 6-day-old *bol-D* plants germinated in 250 µM DIECA. (**f**–**i**) Rosette phenotype of 6-day-old *bol-D* and Ws plants treated with 250 µM DIECA. Scale bar = 0.5 cm (**c**–**e**); 0.25 cm (**f**–**i**). In (**a**,**b**), ANOVA *p*-value = 2 × 10^−16^. Letters represent different statistical groups (LSD test).

We observed that, 9 days after germination (dag), root length and rosette diameter of wild-type plants treated with 50 µM MeJA and *bol-D* plants were equivalent (Figure 3a,b). This could suggest that the reduction in the size of the leaves and root length in *bol-D* plants could be due, at least partially, to an increase in the levels of jasmonates in the plant or another alteration in the pathway.

Despite the reduced size of the wild-type plants upon MeJA application, the characteristic phenotype of *bol-D* plants, which is curvature of the leaves, was not observed; rather, wild-type plants simply reduced their size (Figure 3c).

To test the opposite conditions, we used a previously reported JA biosynthesis inhibitor, sodium diethylditiocarbamate (DIECA) [37]. Interestingly, when *bol-D* plants were grown in the presence of DIECA, the curvature of the leaves was reduced, suggesting that this phenotype might result from an alteration in the internal jasmonate balance, which cannot be phenocopied by exogenous JA application (Figure 3d–i). The phenotype may also be the result of the combination of alterations in different processes caused by the gain of *BOL* function, including JA homeostasis.

### 2.4. Changes in BOL Expression Cause Differences in Jasmonic Acid Accumulation

The previous experiments suggested that some of the morphological phenotypes displayed by *bol-cr* and *bol-D* might be, at least partially, due to alterations in jasmonate homeostasis. Therefore, we quantified the level of jasmonic acid in different genetic backgrounds: (i) the Arabidopsis gain-of-function mutant *bol-D*; (ii) a BOL inducible line (*35S::ESR2-ER*), in which BOL is fused to a β-estradiol receptor fragment, and the application of β-estradiol (β-est) promotes its entrance to the nucleus [4,38]; (iii) a *35S::BOL* overexpressor line in tobacco; and (iv) Arabidopsis *bol-cr2* inflorescences. These were compared to their respective wild-type counterparts. For the inducible line, JA content was also compared to non-induced control plants, and it was evaluated two and four days after induction.

**Figure 4:**
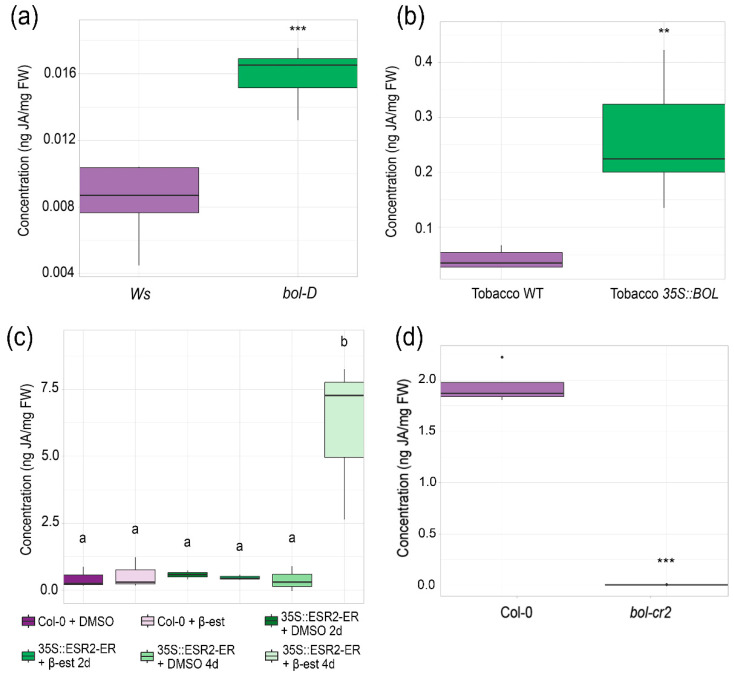
Jasmonic acid content analyses in BOL gain and loss-of-function mutants and BOL constitutive and inducible overexpression lines. Quantification of jasmonic acid in (**a**) Ws wild-type and *bol-D* plants (Student’s *t*-test, *p*-value = 0.0004) and (**b**) wild-type and *35S::BOL* tobacco (Student’s *t*-test, *p*-value = 0.002), (**c**) wild-type Col-0 and *35S::ESR2-ER* plants two and four days after induction (ANOVA *p*-value = 0.0007; letters represent different statistical groups; LSD test), and (**d**) wild-type Col-0 and *bol-cr2* inflorescences (Student’s *t*-test, *p*-value = 9.07 × 10^−7^, asterisks denote significance levels: ** *p* < 0.01, *** *p* < 0.001).

Interestingly, BOL gain-of-function and overexpressor plants were found to contain more jasmonic acid than wild-type plants. Arabidopsis *bol-D* and tobacco *35S::BOL* plants presented a 2-fold and 6-fold increase, respectively, in JA content by mg of fresh weight than their corresponding wild-type plants (Figure 4a,b). In the inducible BOL line *35S::ESR2-ER*, a higher JA content was detected 4 days after induction compared to non-induced plants (Figure 4c). Conversely, when comparing the JA content in inflorescences of wild-type and *bol-cr2* mutant plants, *bol-cr2* presented a reduction, containing less jasmonic acid than wild-type Col-0 plants (Figure 4d).

These results suggest that the jasmonate level is altered when BOL expression or function is altered.

### 2.5. BOL Affects the Expression of the JA Response Marker pJAZ10::GUSPlus

**Figure 5:**
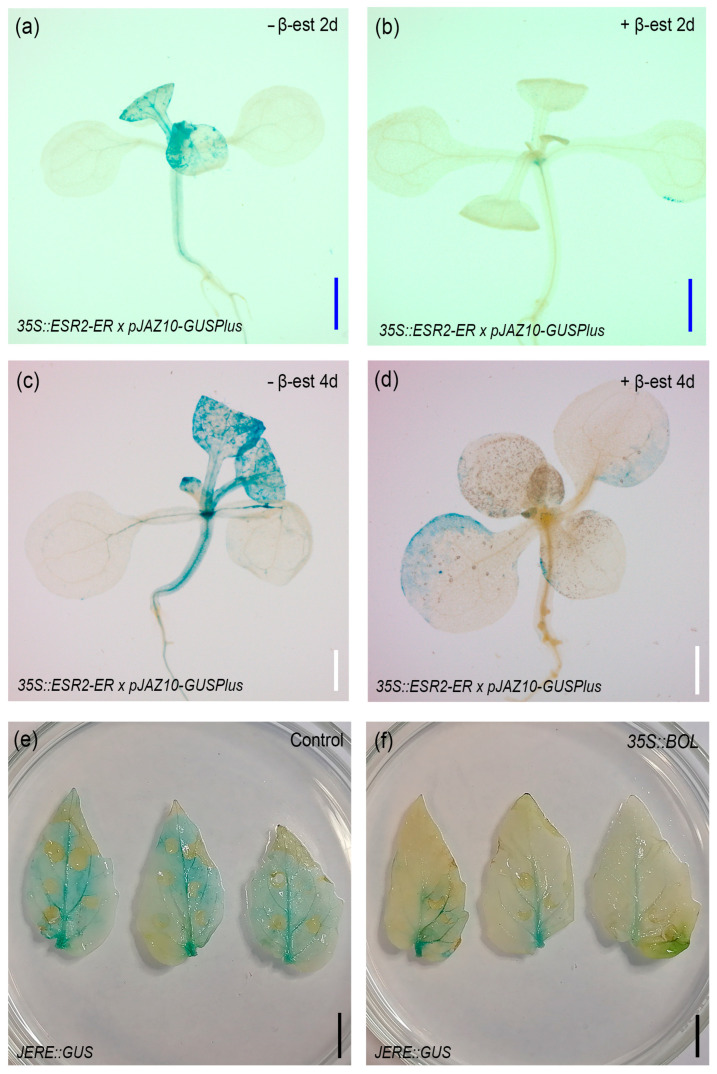
JA reporter line expression after BOL induction or transient expression. (**a**–**d**) Expression of the Arabidopsis JA reporter *pJAZ10::GUSPlus* in the *35S::ESR2-ER* BOL inducible background, transferred to mock (**a**,**c**) or induction media supplemented with β-estradiol (β-est) (**b**,**d**), 2 (**a**,**b**) and 4 (**c**,**d**) days after transference. (**e**,**f**) Expression of the tomato JA reporter *4xJERE::GUS* after agroinfiltration with control (**e**) or *35S::BOL* construct (**f**), evaluated 4 days after agroinfiltration. Scale bars = blue 1 mm (**a**,**b**), white 0.5 mm (**c**,**d**), black 1 cm (**e**,**f**).

After observing the changes in JA content, we explored the effect of increased BOL activity in the transcriptional response to JA. For this, we analyzed the expression of the JA response marker *pJAZ10::GUSPlus* [39] in the inducible *35S::ESR2-ER* background. We transferred 9-day-old seedlings to a β-estradiol medium (inducing medium) and analyzed the expression of the response marker from 1 to 4 days after induction. In normal conditions, expression was detected in the hypocotyl and meristematic region of the seedlings, and patchy expression was observed in the cotyledons and developing leaves (Figure S2a,c,e,g). At 2 and 4 days after BOL induction, we observed a reduction in expression in the hypocotyl and meristematic region (Figure 5b,d and Figure S2d,h) compared with those plants that were not induced (Figure 5a,c and Figure S2c,g). This expression pattern was consistently observed across the 1 to 4 days of analyses (Figure S2). We also evaluated the expression of the tomato JA response marker *4xJERE::GUS* [40] after the transient expression of a *35S::BOL* construct. We observed that the leaves that were infiltrated with the mock solution presented GUS expression in almost all the leaf (Figure 5c), but the ones infiltrated with the *35S::BOL* construct presented a reduced expression of the marker (Figure 5d). These results suggest that the transient or inducible increased expression of *BOL* can cause a reduction in the response to JA, at least in the analyzed times and tissues.

### 2.6. Jasmonate Inhibition Impairs the Formation of BOL-Induced Green Calli

**Figure 6:**
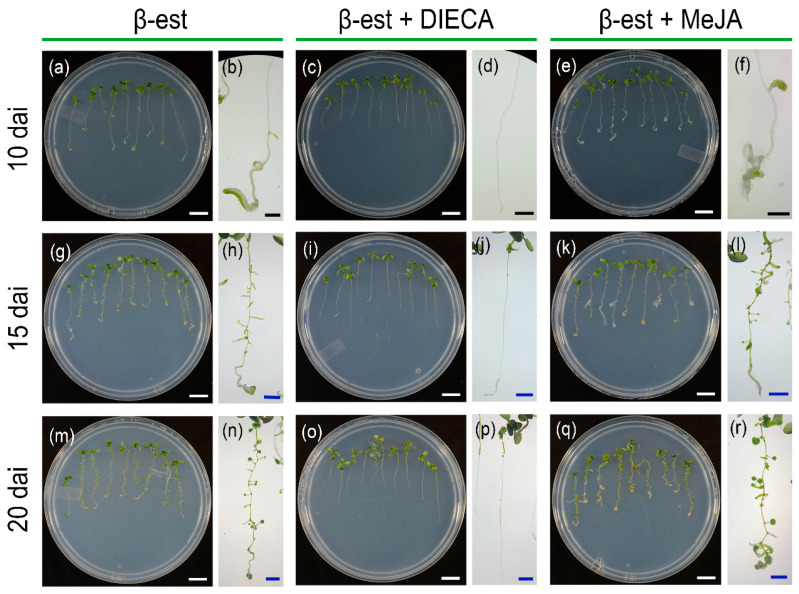
Effect of jasmonate inhibition or exogenous application in BOL-induced green calli formation. Induced *35S::ESR2-ER* plants in medium supplemented with only 10 µM β-estradiol (β-est) (**a**,**b**,**g**,**h**,**m**,**n**), in medium supplemented with 10 µM β-est and 250 µM DIECA (**c**,**d**,**i**,**j**,**o**,**p**), or in medium supplemented with 10 µM β-est and 50 µM MeJA (**e**,**f**,**k**,**l**,**q**,**r**), 10 (**a**–**f**), 15 (**g**–**l**), and 20 (**m**–**r**) days after induction (dai). Scale bars = white 1 cm, black 1 mm, blue 2.5 mm. MeJA, Methyl Jasmonate; DIECA, sodium diethyldithiocarbamate (JA biosynthesis inhibitor).

One of the characteristic phenotypes of *BOL* increased activity is the spontaneous formation of green calli in roots without the addition of any hormones. We asked whether the application of JA or the JA biosynthesis inhibitor DIECA would inhibit or exacerbate this phenomenon. For this, we analyzed the formation of green calli in inducible *35S::ESR2-ER* plants cultivated in medium supplemented with MeJA or DIECA. We observed that regions with green calli were visible 10 days after induction, mostly at the tip of the root, in control plants and those treated with MeJA (Figure 6a,b,e,f and Figure S3a,c). However, these regions were less evident in the plants that were treated with JA synthesis inhibitor (Figure 6c,d and Figure S3b). Further in time, 15 and 20 days after germination, the green calli areas were observed along the entire root in the mock and MeJA-treated induced plants (Figure 6g,h,k–n,q,r and Figure S3d,f,g,i). In contrast, the plants treated with DIECA only presented small green calli areas in the differentiated part of the root, at the top of the root regions located close to the hypocotyl (Figure 6i,j and Figure S3e,h). These results suggest that the inhibition of JA biosynthesis reduced the ability of BOL/ESR2 to promote callus formation in roots, and they may indicate that JA is required for the formation of green calli induced by BOL/ESR2 activation.

### 2.7. The Inhibition of JA Biosynthesis Alters the Pattern of BOL Expression

To explore whether JAs could also play a role in the indirect regulation of *BOL* expression, we analyzed the expression of the *BOL* reporter line *pBOL::GUS* [2] in plants treated with and without the JA biosynthesis inhibitor.

**Figure 7:**
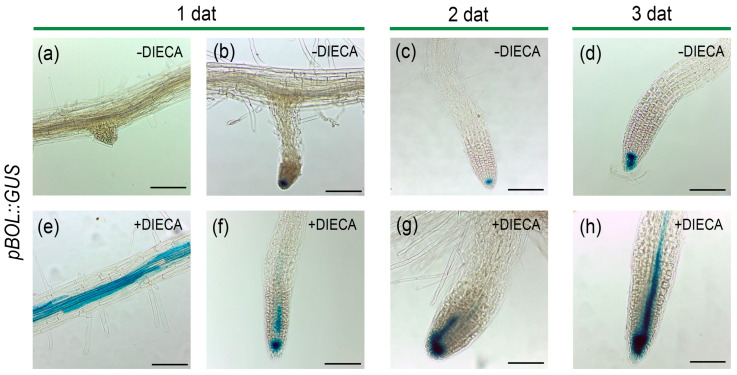
*BOL* expression in plants treated with a JA synthesis inhibitor. *BOL* expression in *pBOL::GUS* plants in mock medium, showing sporadic expression at the root tips (**a**–**d**) and plants in medium supplemented with 250 µM DIECA (**e**–**h**), one (**a**,**b**,**e**,**f**), two (**c**,**g**), and three (**d**,**h**) days after treatment (dat), where GUS staining is observed in all roots analyzed and expands to the region above the root tip and differentiated tissue. Scale bars = 0.1 mm.

As previously reported, in control seedlings we observed the expression of *BOL* in leaf primordia [2,4]. We also observed sporadic expression in meristematic regions of the main and lateral roots (Figure 7a–d). Interestingly, when plants were treated with DIECA, we observed that the *BOL* expression domain expanded in the roots. From the only sporadic expression at the root tips, the expression was now observed in all roots and had expanded to the region above the root tip.

The expansion in the expression domain was larger as the plants were exposed to the JA inhibitor for longer times (Figure 7f–h). We also observed the ectopic expression of *BOL* in epidermal cells of the differentiated zone of the roots (Figure 7e). These changes suggest that JA appears to restrict *BOL* expression.

## 3. Discussion

The main question of this study was to explore the connections between the BOL/ESR2/DRNL transcription factor and the jasmonate pathway. The results of this exploration indicate that BOL can modulate the jasmonate pathway and that JAs restrict *BOL* expression.

The phenotypes of loss-of-function mutants include defects in floral organs, particularly stamens and gynoecia [3,5,7]. JA plays an important role in stamen development, as mutants of some of the JA biosynthesis enzymes present male sterility in Arabidopsis due to deficient filament elongation, non-viable pollen, and delayed anther dehiscence [22,29,41,42,43]. Defects in floral organs and sterility in JA biosynthetic gene mutants have also been reported in other species such as rice, tomato, or maize, highlighting the importance of JA in floral organ development [44]. Our results showed that an exogenous MeJA application could partially recover the wild-type phenotype in the *bol-cr2* mutant, suggesting that a part of the *BOL* gene function in floral organ development involves achieving or maintaining proper JA homeostasis. One of the possible reasons why only partial recovery of the phenotype could be achieved and not a complete recovery is that BOL may be coordinating different processes simultaneously, including different hormonal pathways, not only jasmonates. Floral development also involves other hormones, such as auxins and gibberellins [45,46,47]. Another possibility is that, in addition to affecting the jasmonate content, the loss-of-function mutation also affects the response to this phytohormone so that, despite the increased content, the mutant becomes unable to respond in the same way as a wild-type plant. Surprisingly, we were only expecting partial recovery of organ formation, but a reduction in organ fusion was also observed. To date, we have not found information about a possible role of jasmonates in preventing organ fusion, and this is an interesting observation to further explore.

JA application or inhibition in wild-type or dominant mutants, and JA content quantification, further revealed a connection between BOL and this hormonal pathway. When we treated wild-type plants with MeJA, we observed a reduction in rosette size, which resembled the size of *bol-D* rosettes. JAs affect the cell cycle, and they inhibit cell growth in leaves [32]. Jasmonates affect cellular proliferation and expansion, and they could play a role in the synchronization of the development of different tissues in a new organ at specific stages. We noticed that the addition of exogenous jasmonates to wild-type plants caused a reduction in rosette size without leading to the leaf curvature characteristic of *bol-D* plants. However, when *bol-D* plants were treated with a jasmonate inhibitor, DIECA, leaf curvature was reduced, leading to flatter leaves, more similar to wild-type leaves. Therefore, even when the MeJA application could not replicate the curved leaf phenotype in wild-type plants, the inhibition of JA biosynthesis recovered the wild-type phenotype. This could suggest that the phenotype of the *bol-D* mutant could be caused, at least partially, by an excess of jasmonates in the plant, either one of the active molecules of the jasmonate pathway or a modified jasmonate molecule that is only produced in high levels in the *bol-D* mutant, possibly due to upregulation of JA biosynthesis genes. Another scenario is that jasmonates within the *bol-D* mutant plant could have a different distribution than in wild-type plants. Moreover, the leaf curvature phenotype may require a combination of changes in other pathways or genes, together with JAs, which may occur in the *bol-D* mutant but not in wild-type plants. Exploring this in greater detail through additional research will be essential to elucidate *BOL* function and can provide insights into the mechanisms by which it affects leaf shape.

Another phenotype that was recovered by the inhibition of JA biosynthesis was the spontaneous formation of green calli in roots, a conspicuous phenotype produced by BOL induction. Callus formation was severely reduced when plants were treated with DIECA, suggesting that jasmonates may act downstream of BOL or are required for the BOL-promoted callus formation phenotype. This finding was unexpected because these calli appear to develop from lateral roots, and calli have been found to have lateral root primordia identity [48,49]. JAs are known to inhibit lateral root formation [50,51]. However, the inhibition of JA biosynthesis by DIECA severely reduced the formation of calli. Therefore, the role of JA and the effect of DIECA on callus formation in induced *35S::ESR2-ER* plants may not be due to an effect on lateral root formation, but rather in the conversion to green callus, possibly. In this work, we did not explore other phenotypes that may have been recovered by JA biosynthesis inhibition, and it will be interesting to explore further.

BOL seems to affect both JA levels and response. The phenotypes obtained after the JA and inhibitor treatments suggested that some of the changes in the development of *BOL* gain- and loss-of-function mutants, and inducible line, could be caused by an altered level of jasmonates. When JA levels were analyzed, we observed that all BOL overexpressor plants (*35S::BOL* in tobacco, *bol-D* and *35S::ESR2-ER* in Arabidopsis) contained more jasmonic acid than wild-type plants, in contrast to the *bol-cr2* mutant, in which JA levels decreased compared to wild-type plants. This indicated that BOL is able to modulate JA levels. Curiously, when BOL was induced in JA reporter lines, they showed reduced expression. This effect might be caused by a compensation where the increased JA accumulation by BOL induction activates *JAZ* genes as a part of a negative feedback loop in JA signaling, resulting in the reduced JA response observed despite the high levels of the hormone. Furthermore, the timing and tissue type in which these effects are exerted may be different. Future experiments will be essential to evaluate the JA response and accumulation upon BOL activation in different tissues and stages to better understand the temporal and spatial dynamics of this effect or regulation.

At this moment, these experiments do not reveal whether the influence of BOL on the jasmonate pathway is direct, with BOL binding and regulating the expression of JA genes, or indirect, through the regulation of other genes that in turn affect the pathway. It will be interesting to explore this further. It could be indirect, since a clear increase in JA content in inducible BOL *(35S::ESR2-ER)* occurred four days after induction. Recently, we found that BOL/ESR2 causes an increase in cytokinin content [12], and cytokinin has been reported to promote JA accumulation [52]. This could also be happening in Arabidopsis, but we cannot discard the possibility of direct regulation of BOL upon JA-related genes. Nevertheless, regardless of whether the regulation is direct or indirect, it may not be involved in rapid responses to wounding or stress, since these stimuli do not appear to activate *BOL* transcription.

However, JAs play a “double role” in the plant as chemical signals that trigger a rapid plant response in defense to herbivory [53,54] and wounding [55] and as developmental signals. In this regard, JA genes are also developmentally regulated. For instance, the transcription factor AGAMOUS (AG) directly regulates the expression of the JA biosynthetic gene *DEFECTIVE IN ANTHER DEHISCENCE 1* (*DAD1*), ensuring proper JA levels during stamen maturation, including elongation of the filaments, pollen maturation, and anther dehiscence [47]. As in this example, we speculate that the role of BOL in the JA pathway may be related to developmental regulation.

Curiously, the interaction between the BOL and the JA pathways appears to include a feedback loop, as JAs appear to negatively affect *BOL* expression. The *BOL* expression pattern has been reported to be restricted to aerial developing young tissues and organs [2,3,4,5]. We have observed that this pattern is constant and remains unchanged in several different conditions. Interestingly, the expression pattern clearly changed when JA biosynthesis was inhibited. This was evident in the root. Though *BOL* expression is constant in very young developing aerial organs, it is also sporadically expressed in the tips of the main and the young lateral roots. This expression occurs only in very few roots, is not constant, and is very low [2]. The presence of low counts of *BOL* transcripts in RNA-seq experiments [56] and the longer root phenotype in *drnl-2* and *bol-cr* loss-of-function mutants suggest that the gene could be expressed at very low or transient levels in specific regions of the roots, though this has not yet been thoroughly investigated.

The effect of JA inhibition on *BOL* expression in the root was striking. When the reporter line was transferred to a medium supplemented with the JA biosynthesis inhibitor, expression was then observed in all the analyzed roots and expanded to reach the inner tissues above the tip of the root, and even some mature root regions, where it was not observed before. This suggests that normal levels of JA, either in a cell or non-cell autonomous way, could be repressing *BOL* expression in the root. This may point to a feedback loop in the BOL–JA interaction; as BOL seems to modulate JA signaling and accumulation, JA, in turn, restricts *BOL* expression. It will be interesting to determine whether this effect is reflecting a repressing regulatory role of the JA pathway in *BOL* expression, and if so, whether this regulation is directly exerted by transcription factors downstream of the pathway (such as MYC2) or indirectly by affecting other factors. If it were a direct consequence of reduced jasmonate content within the plant, it could be a feedback loop, whereby, to restore the normal level of jasmonates, *BOL* gene expression is promoted. However, more experiments are needed to corroborate these speculations. When searching for elements that can regulate *BOL* and *LFS* expression, both *BOL* and *LFS* promoters were found to include GCC boxes [8], which are well-characterized cis-regulatory elements involved in the transcriptional regulation of jasmonate-responsive genes through their interaction with ERF transcription factors [57]. This could suggest that BOL expression may be directly modulated by jasmonates, but more research is required to test whether this is the case.

With the results presented in this work, we propose that BOL modulates the jasmonate pathway in a developmental context. Future experiments will be crucial to determining whether the regulation of the JA pathway by BOL is direct or indirect, identifying the specific JA pathway genes or intermediary genes targeted by BOL, and shedding light on the biological relevance of this regulation. Moreover, the putative regulatory JA–BOL feedback loop should be further studied to determine the spatiotemporal dynamics of this regulation, which will provide deeper insights into the mechanisms underlying the BOL and JA pathway interaction and its biological role.

## 4. Materials and Methods

### 4.1. Plant Materials

The lines used in this study were: Columbia (Col-0), Landsberg *erecta* (L*er*), and Wassiliewskija (Ws) wild-type ecotypes; *bol-D* [2], *drnl-2* [5], and *bol-cr1* and *2* (generated in this work) mutants; *pJAZ10::GUSPlus* (JA response marker line) [39]; *pBOL-GUS* (*BOL* marker line) [2]; the inducible overexpression *BOL* line *35S::ESR2-ER* for *Arabidopsis thaliana* [4,38]; *35S-BOL* (*BOL* overexpressor line) and wild-type tobacco (*Nicotiana tabacum* cv SR1) [2]; and the jasmonate response marker line, *4xJERE::GUS* in tomato (cv MT), where the tomato jasmonic acid response element (AGACCGCC) is fused to the β-glucuronidase gene [40].

### 4.2. Plant Growth

Arabidopsis seeds were sown in soil (peat moss, perlite, vermiculite 3:1:1) and were kept at 4 °C for 2 days before being transferred to a growth chamber at 22 °C under long day conditions. Two weeks after germination, plants were transferred to a greenhouse with natural light and temperature conditions. Tobacco and tomato seeds were grown in soil (peat moss, leaf soil, lama soil, perlite, and vermiculite 3:2:1:1:1) in a growth room at 24 °C under long day conditions (16 h of light/8 h of dark).

For Arabidopsis in vitro analyses, seeds were disinfected with chlorine gas in a vacuum chamber for 5–6 h and were sown in medium containing 0.5× MS (Caisson Labs, TX, USA), 1% sucrose, and 1% agar. Plates were kept for 2 days at 4 °C before being transferred to a growth chamber at 22 °C under long day conditions. Plates were incubated vertically for root measurement and calli evaluation and horizontally for rosette measurement.

### 4.3. Generation of a CRISPR-Cas9 Induced New Allele of BOL

Guide RNAs (gRNAs) were designed with the bioinformatic tool CRISPR-P [58]. Guides flanking the region coding for the AP2 domain in *BOL*, with the best score and without off-targets, were selected.

The vector containing the Cas9/gRNA cassette was generated using the Golden Gate system vectors [59,60]. gRNAs were inserted individually into level 1 vectors pICH47751 (gRNA1) and pICH47761 (gRNA2). Both gRNA constructs, NOSp::NPT2 OCST (selection marker; pICH47732) and pRPS5A:Cas9-NOSt (pICH47742) were inserted into the level 2 binary vector pAGM4723.

For the synthesis of the RNA fragments using vector pICH86966::AtU6p::sgRNA_PDS as a template, the PCR conditions were: 94 °C—5 min; 94 °C—15 s, 60 °C—30 s, 68 °C—1 min, 30 cycles; 68 °C—5 min. Purification of PCR fragments was performed with “DNA Clean & ConcentratorTM-5 Kit” (Zymo Research, CA, USA). Level 1 reaction conditions were: 37 °C—3 min, 16 °C—4 min, 20 cycles; 50 °C—5 min; 80 °C—10 min. Level 2 reaction conditions were: 37 °C—8 min, 16 °C—8 min, 7 cycles; 37 °C—10 min; 80 °C—15 min. Plasmid extraction was performed with “ZyppyTM Plasmid Miniprep Kit” (Zymo Research, Irvine, CA, USA). The *Bsa*I and *Bpi*I enzymes (BioLabs, San Diego, CA, USA) were used for digestion of level 1 and level 2 vectors, respectively. The T4 DNA ligase (Promega, Madison, WI, USA) enzyme was used for ligation. The final vector was corroborated by colony PCR, digestion with *BamH*I, *EcoR*I (Fermentas, Waltham, MA, USA), *Pst*I (Thermo SCIENTIFIC, Waltham, MA, USA), *Afi*II (BioLabs, MA, USA) enzymes, and, finally, by sequencing.

The binary vector with the final construct was transformed in *A. tumefaciens* GV3101, and Col-0 plants were transformed by floral dip [61]. T0 transformant plants were selected with kanamycin. T1 and T2 transformants were genotyped by PCR and finally by sequencing. All the primers used are listed in Table S1.

### 4.4. GUS Expression Analysis

GUS staining was performed at 37 °C overnight with a standard X-gluc solution [3]. Images were taken with a Leica ICC50 HD optical microscope (Leica Microsystems, Wetzlar, Germany).

### 4.5. Phenotypic Analyses and Documentation

Images of floral organs of Col-0, *bol-cr1* and *bol-cr2* were taken using a Keyence VHX-7000 digital microscope (Keyence Corporation, Osaka, Japan). Pictures of in vitro-grown plants were taken with a Zeiss Stemi 2000-C stereoscope (Carl Zeiss AG, Jena, Germany) and a Nikon D7100 camera, (Nikon Corporation, Tokyo, Japan) and they were analyzed with ImageJ 1.54g software.

### 4.6. BOL Transient Expression Assays

*Agrobacterium tumefaciens* (GV2260) harboring a pMDC204 vector with the *35S::BOL* construct was grown in 5–10 mL of LB medium supplemented with kanamycin (50 mg/L), rifampicin (25 mg/L), and ampicillin (50 mg/L) at 28 °C for 2 days until saturation (OD > 1). The culture was centrifuged at 4000 rpm for 10 min at room temperature, and the collected cells were resuspended in 10 mL of infiltration medium (10 mM MgCl_2_, 10 mM MES pH = 5.6, 100 mM acetosyringone). The bacteria were left in slight agitation at room temperature for at least 3 h. The cell suspension was injected into the abaxial side of young leaves of *4xJERE-GUS* tomato plants with a 3 mL syringe without needle. Four to five injections were made in leaves of the same developmental stage of five different 8-week-old plants. Infiltrated leaves were collected 4 days after infiltration to evaluate GUS activity. Infiltration medium without bacteria was used as a mock treatment.

### 4.7. MeJA and DIECA Treatments

Before the experiment, new inflorescences emerging above cauline leaves of 6-week-old plants were identified and marked. They were allowed to grow for 8 days, and then all their open flowers were removed. The remaining closed flower buds were sprayed with a MeJA solution (50 or 100 μM MeJA, 0.01% Silwet L-77 in distilled water) in the morning and in the afternoon of that day. Starting one day after the spraying, 25 open flowers were collected each day, and the rest of the open flowers were removed. Staminoid structures of the collected flowers were analyzed. This procedure was performed for seven consecutive days to evaluate buds that were at different stages of development at the moment of treatment.

For the in vitro experiments, plants were grown in Petri dishes with MS and agar medium (as previously described) supplemented with either 50 µM MeJA or 250 µM DIECA.

### 4.8. Jasmonic Acid Quantification

Jasmonic acid was extracted from 250 mg of fresh tissue and analyzed according to [62,63,64,65,66] with some modifications. A total of 1 mL of ethyl acetate and 10 μL of 0.1 mg/mL (*±*)-Dihydrojasmonic acid (internal standard; Cat: CDS022683, Sigma-Aldrich, St. Louis, MO, USA) were added to each sample and mixed in a vortex. The samples were agitated for 30 min, and the supernatant was mixed with the previous one. The solvent was completely evaporated with nitrogen gas, and 100 μL of N′N′ di-isopropyl-ethylamine, 100 μL of chloroform, and 10 μL of PFB-Br (2,3,4,5,6-pentafluorobenzyl bromide, Sigma-Aldrich) were added to each sample for derivatization. The samples were incubated at 60 °C for 30 min. The solvents were completely evaporated with nitrogen gas, and the samples were resuspended in 200 μL of methanol, HPLC grade. A total of 1 µL of each sample was injected to analyze JA using GC-EIMS (Gas Chromatography, Agilent Technologies 7890A-Electron Ionization Mass Spectrometry, Agilent Technologies 5975C, Santa Clara, CA, USA) in SIM mode for 141, 181, 390 *m*/*z*. The separation was performed in a DB-1 MS ultra-inert capillary column (60 m length × 0.25 mm internal diameter and 0.25 μm film thickness; Agilent Technologies). The gas chromatograph was run under the following program: initial temperature 150 °C for 3 min, then a ramp at 4 °C per min up to 260 °C; this temperature was kept constant for 25 min. The quantification of the JA was performed using the internal standard of dihydrojasmonic added to each sample, as indicated above. An average of the areas of the internal standard was obtained for all the samples, obtaining a correction factor by dividing the area of the internal standard by the area of each sample and multiplying it by the correction factor obtained for each sample. The average area of the internal standard corresponds to a concentration of 0.005 µg/µL.

### 4.9. Statistical Analysis

Statistical analyses and graphs were made using RStudio (Version 4.2.0, RStudio Team, Boston, MA, USA). For parametric variables, ANOVA or Student’s *t*-tests were used, while the Kruskal–Wallis test was employed for nonparametric variables. When significant differences were found, LSD and Dunn’s post hoc tests were applied.

## Data Availability

Data are contained within the article and Supplementary Materials.

## Supplementary Materials

The following supporting information can be downloaded at: https://www.mdpi.com/article/10.3390/plants14121757/s1; Figure S1. Genotyping of *bol-cr* mutants by PCR using genomic DNA; Figure S2. JA reporter line expression changes upon BOL induction; Figure S3. Effect of jasmonate inhibition or exogenous application in BOL-induced green calli formation; Table S1. Primers used for the generation of CRISPR-Cas9-induced BOL alleles *bol-cr1* and *bol-cr2*.
