# Supplementary material for: Interactions Between the Transcription Factor BOL/DRNL/ESR2 and the Jasmonate Pathway
Source: Plants (Basel). 2025 Jun 8;14(12):1757. doi: 10.3390/plants14121757 (PMC12196909; doi:10.3390/plants14121757)
Supplement: Supplementary file 1 [file plants-14-01757-s001.zip › plants-3657245-supplementary.pdf]

Supplementary Material

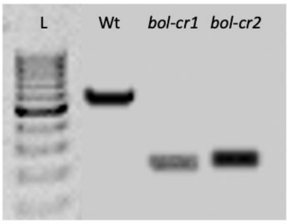

Figure S1. Genotyping of *bol-cr* mutants by PCR using genomic DNA. In the electrophoresis gel, smaller bands in *bol-cr* mutants are caused by a deletion in the *BOL* AP2 domain (wt). The deletion was confirmed by sequencing. L: DNA size ladder.

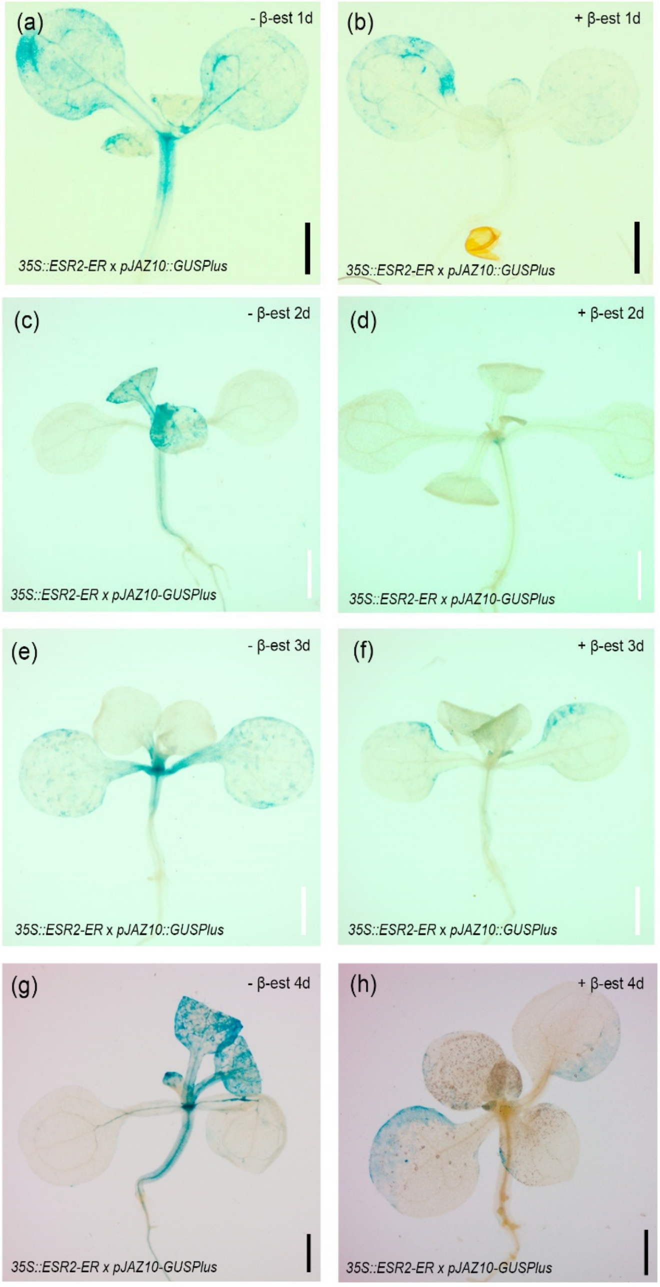

Figure S2. JA reporter line expression changes upon BOL induction Expression of the *pJAZ10::GUSPlus* marker in the inducible line background, transferred to mock (a, c, e, g) and induction media (b. d, f, h) 1 (a, b), 2 (c, d) 3 (e, f) and 4 (g, h) days after transference. Scale bars = black 1 mm, white 0.5 mm.

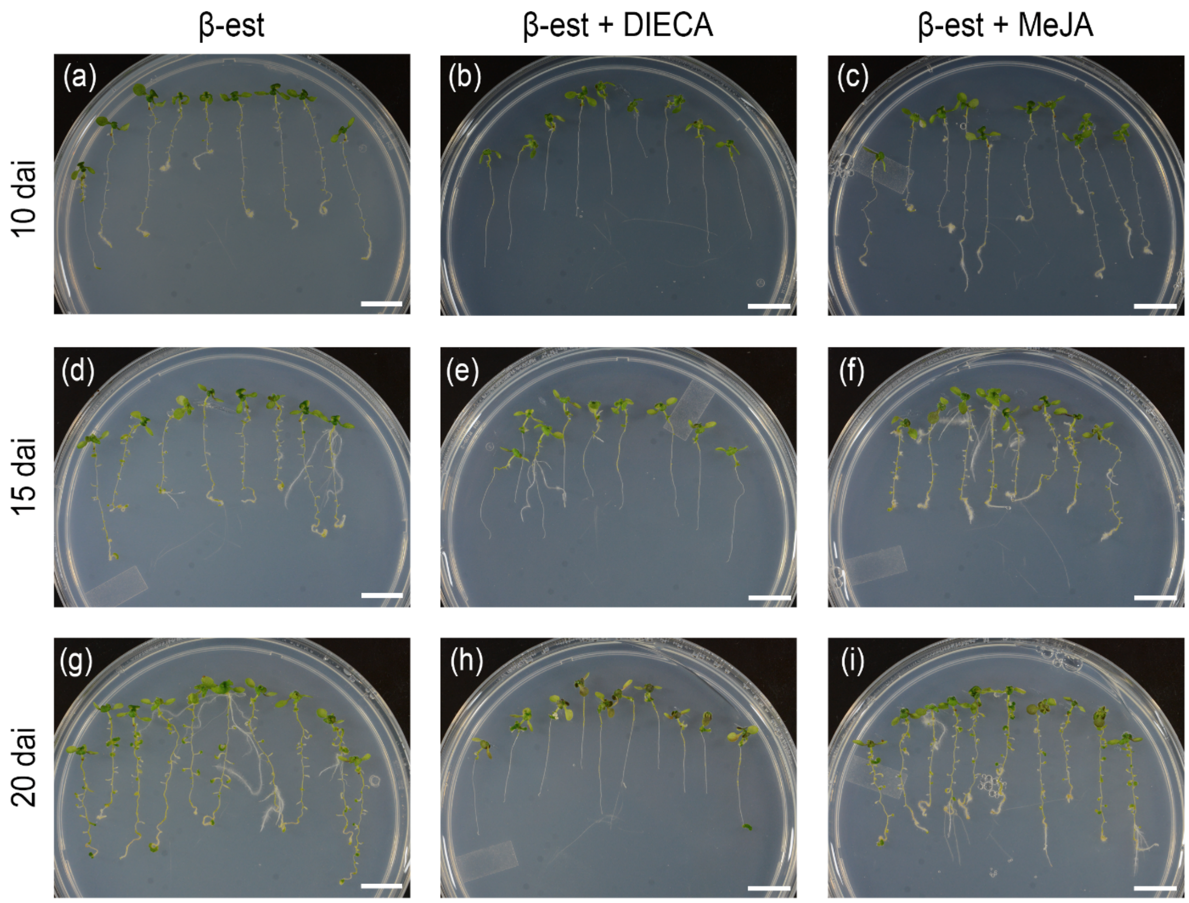

Figure S3. Effect of jasmonate inhibition or exogenous application in BOL-induced green calli formation. Induced *35S::ESR2-ER* plants in control medium, with green calli (a, d, g), or in medium supplemented with 250 µM DIECA (b, e, h) or 50 µM MeJA (c, f, i) at 10 (a-c), 15 (d-f) and 20 (g-i) days after induction. Scale bar = 1 cm.

Table S1. Primers used for the generation of CRISPR-Cas9 induced BOL alleles *bol-cr1* and *bol-cr2*.

Primer	Sequence	Comment
gRNA U RV	tgtggtctca**AGCG**TAATGCCAACTTTGTAC	Universal reverse primer to amplify gRNA + scaffold (blue: restriction site for BsaI; bold: overhang)
BOL gRNA1	tgtggtctca**ATTG**AGGAGACGAGAAAATACGG GTTTTAGAGCTAGAAATAGCAAG	To sintesize BOL gRNA (blue: restriction site for BsaI; bold: overhang; red: gRNA)
BOL gRNA2	tgtggtctca**ATTG**TACCTTATCGTAGAGACACG TTTTAGAGCTAGAAATAGCAAG	
U6-26 FW	GGCCCCTGGGAATCTGAAAG	To verify level 1 constructs
pAGM4723 FW	GATTTTGTGCCGAGCTGCCG	To verify level 2 constructs
pAGM4723 RV	CGCACGGCTGGCACATACAA	
Cas9 FW	TCGAACTCGAGAACGGTAGAA	To amplify Cas9
Cas9 RV	CAAGACCGGCAACAGGAT	
BOL FW	GTGCCTCACAACCCTAAGAC	To genotype CRISPR induced BOL mutants
BOL RV	GCGGGTAAGCGAAAGTGGTT	
